# Hepatic Bone Morphogenetic Protein and Activin Membrane-Bound Inhibitor Levels Decline in Hepatitis C but Are Not Associated with Progression of Hepatocellular Carcinoma

**DOI:** 10.3390/biomedicines12102397

**Published:** 2024-10-19

**Authors:** Florian Weber, Kirsten Utpatel, Katja Evert, Thomas S. Weiss, Christa Buechler

**Affiliations:** 1 Institute of Pathology, University of Regensburg, 93053 Regensburg, Germany; florian.weber@klinik.uni-regensburg.de (F.W.); kirsten.utpatel@klinik.uni-regensburg.de (K.U.); katja.evert@klinik.uni-regensburg.de (K.E.); 2 Children’s University Hospital (KUNO), Regensburg University Hospital, 93053 Regensburg, Germany; thomas.weiss@ukr.de; 3 Department of Internal Medicine I, Regensburg University Hospital, 93053 Regensburg, Germany

**Keywords:** histologic grade, fibrosis, hepatitis, UICC score, immunohistochemistry

## Abstract

Background/Objectives: Bone morphogenetic protein and activin membrane-bound inhibitor (BAMBI) is an antagonist of transforming growth factor (TGF)-β type 1 signaling. BAMBI functions as an anti-fibrotic protein and exerts pro- as well as anti-cancerogenic activities. Our study aimed to correlate hepatocyte BAMBI protein levels in hepatocellular carcinoma (HCC) with T stage, lymph node invasion, vessel invasion, grading, tumor size and Union for International Cancer Control (UICC) stage, as well as with liver inflammation and fibrosis stages. Methods: Hepatocyte BAMBI protein expression was assessed by immunohistochemistry in HCC tissues of 320 patients and non-tumor tissues of 51 patients. Results: In the HCC tissues of the whole cohort and sex-specific analysis, BAMBI protein was not related to T stage, vessel invasion, lymph node invasion, histologic grade, UICC stage and tumor size. Accordingly, BAMBI was not associated with overall survival, recurrence-free and metastasis-free survival. BAMBI protein levels in tumor and non-tumor tissues were not related to inflammation and fibrosis grade. BAMBI protein levels in HCC tissues and non-tumor tissues from HCC patients, which were analyzed by immunoblot in a small cohort and by immunohistochemistry in the tissues of patients described above, were similar. Notably, BAMBI protein was low-abundant in HCC tissues of hepatitis C virus (HCV) compared to hepatitis B virus (HBV)-infected patients with comparable disease severity. Immunoblot analysis revealed reduced BAMBI protein in non-tumor tissues of patients with HCV in comparison to patients with HBV and normal human liver tissues. Conclusions: In summary, this analysis showed that hepatocyte BAMBI protein levels of patients with HCC are related to HCV infection rather than the severity of the underlying liver disease and cancer staging.

## 1. Introduction

Bone morphogenetic protein and activin membrane-bound inhibitor (BAMBI) is a pseudoreceptor that can associate with transforming growth factor (TGF)-β type 1 receptors to inhibit the formation of the receptor complex and the signaling of ligands such as TGF-β [1,2]. The TGF-β family of cytokines includes TGF-β, bone morphogenetic proteins (BMPs) and activins [2,3]. TGF-β is a well-studied cytokine and regulates inflammation, fibrogenesis and proliferation [4,5,6,7]. Hepatic BAMBI levels are reduced in rodent models of liver injury and in the fibrotic liver of patients [8,9,10,11]. Downregulation of the expression of BAMBI is thought to enhance the various effects of TGF-β [1,2] and low BAMBI was associated with increased activation of hepatic stellate cells and fibrogenesis [8,9,10,11].

Early studies indicated that BAMBI is specifically expressed in hepatic stellate cells [9,12]. It was later shown that hepatocytes also express BAMBI [10]. Compared to hepatic stellate cells, the function of BAMBI in hepatocytes is much less well understood [2].

Chronic hepatitis B virus (HBV) and hepatitis C virus (HCV) infections, alcohol abuse and obesity are leading risk factors for liver fibrosis [13,14,15]. Chronic liver diseases are associated with a higher risk of hepatocellular carcinoma (HCC) [16,17,18]. BAMBI blocks TGF-β activity, which normally induces cell growth arrest [19], and in cancer cells, high BAMBI expression is thought to promote tumor growth [2,20,21]. However, TGF-β functions as a tumor suppressor in early HCC and contributes to HCC progression in late stages [6]. Consequently, the role of BAMBI may also differ between early and late HCC.

Moreover, BAMBI overexpression protects against liver fibrosis and cirrhosis, which are risk factors for HCC [9,16,22]. Although HCC can develop within a non-cirrhotic liver, it occurs much less frequently than in a cirrhotic liver [23,24].

A few studies having analyzed BAMBI expression in human HCC tissues and function in HCC cell lines reported contradictory results. The high mRNA expression of *BAMBI* in tumors with overexpression of the hepatic stem cell marker, the epithelial cell adhesion molecule, indicated a tumor-promoting function of BAMBI [25]. These poorly differentiated tumors are associated with a shorter overall and disease-free survival [26]. In HBV-positive and HBV-negative patients, the tumor to non-tumor *BAMBI* mRNA ratio was 1.5 and 1.4, respectively, indicating almost similar *BAMBI* mRNA expression in the non-tumor tissues and the tumors [27]. In 53 public datasets, *BAMBI* mRNA was induced in the HCC tissues of 31 cohorts and was similar between the tumor and non-tumor tissues of 22 cohorts [21]. This shows that data from mRNA expression analysis revealed discordant results.

The half-life of *BAMBI* mRNA in murine glomerular endothelial cells is approximately 60 min. In these cells and in hepatic stellate cells, *BAMBI* mRNA was stabilized by blocking protein synthesis with cycloheximide, indicating post-transcriptional regulation [28,29]. In endothelial cells, BAMBI protein is degraded by autophagy [29]. In many situations, transcript levels alone are insufficient to predict protein levels [30] and this may be the case for BAMBI. Therefore, analysis of BAMBI protein levels is required to better understand the role of BAMBI in HCC.

Currently, one study from China has analyzed BAMBI protein in a large cohort of patients with HCC and found that BAMBI protein is highly elevated in HCC tissue compared to non-tumor liver tissue with very low BAMBI protein levels. High BAMBI protein in HCC tissue was associated with shorter overall survival [21].

A study by Zhang et al. showed that overexpression of BAMBI increased the proliferation of Hep3B and Huh7 cells [21]. Nevertheless, experiments analyzing the impact of BAMBI on hepatocyte cell line proliferation also have yielded disparate outcomes [20,31]. Overexpression of BAMBI in HepG2 cells suppressed β-catenin and TGF-β expression and reduced cell proliferation [31].

A study showing that C-terminal-truncated HBV X, which contributes to carcinogenesis, downregulated BAMBI, providing evidence for a tumor-protective role of BAMBI. Xenotransplantation of the BAMBI-expressing HCC cells also revealed a tumor-suppressive role of BAMBI [31]. Overexpression of BAMBI inhibited the TGF-β-driven differentiation of bone marrow-derived mesenchymal stem cells into cancer-associated fibroblasts. The upregulation of tumor-promoting chemokines was blocked by BAMBI [32]. Consistent with these data, BAMBI protein of patients was low in HCC compared to tumor-adjacent tissues. This analysis only used very few patients, which is a limitation of this study [10].

BAMBI has the capacity to bind to six of the seven TGF-β type 1 receptors, and may also block the signaling of ligands other than TGF-β, including bone morphogenetic proteins (BMP) 4 and 6 [2]. The levels of the different TGF-β type 1 receptor ligands and the expression of the respective receptors may vary in the HCC cell lines, and this may contribute to the discordant findings described above.

BMP4 induces BAMBI expression but the physiological relevance of this upregulation has not been clarified [1]. BMP4-deficient mice are not viable, whereas BAMBI loss results in a very mild phenotype [2,33,34,35]. The role of BAMBI in the signaling of the various ligands such as TGF-β, BMPs and activins is understudied and far from being clear [2].

BMP4 increases cell migration and the epithelial–mesenchymal transition, and overexpression of BMP4 in HCC tissues is associated with a poor prognosis [36,37,38,39]. BMP9 is overexpressed in HCC tissues and is a potent tumorigenic factor in the liver [40]. This suggests that BAMBI may not be effective in blocking these increased BMP4 and BMP9 activities in HCC. Possible explanations are that the levels of BAMBI are downregulated in HCC or that the levels of BAMBI do not increase in parallel with the levels of BMPs in HCC. However, data on the expression of BAMBI in HCC are inconsistent [2].

Liver disease etiologies of HCC differ between Asian and Western countries [41]. Chronic HBV is the cause of almost 80% of HCCs in Asia [42]. In Germany HBV, HCV and non-alcoholic fatty liver disease more or less equally contribute to HCC development [43]. However, hepatic BAMBI expression in relation to underlying liver diseases has not been evaluated as far as we know. In addition, ethnic differences in HCC have been described between Asians and Europeans [44,45], and the BAMBI protein has not been studied in a large cohort of European patients. The present study employed immunohistochemistry to determine the BAMBI protein levels in HCC tissues with the objective of identifying associations between the BAMBI protein and the severity of liver disease, tumor size, tumor stage, viral infection and survival in a large cohort of European patients with HCC.

## 2. Materials and Methods

### 2.1. Patients

**Figure 1:**
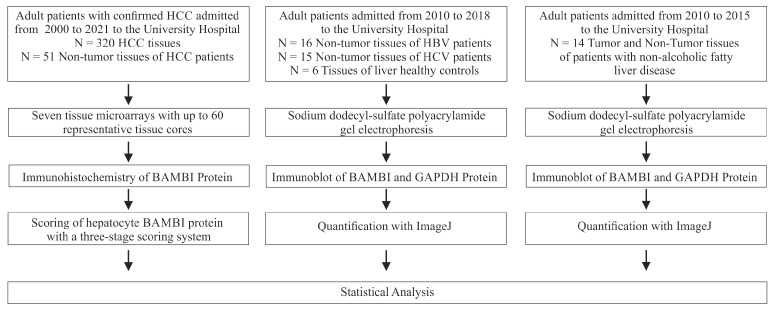
Flow chart of the patients included in the study and the experiments performed.

HCC tissue from 320 patients (262 men and 58 women) was collected between 2000 and 2021 (Figure 1). The patients were from the hospital catchment area, which is mostly the eastern part of Bavaria. The mean age of the patients was 64.4 ± 11.4 years. Non-tumor tissues of 51 HCC patients (7 females and 44 men), who had similar sex distribution, age, T stage, grading, vessel invasion, fibrosis and inflammation scores in comparison to the patients from whom HCC tissues had been collected (*p* > 0.05 for all) were also included (Figure 1).

Seven tissue microarrays (TMAs), containing up to 60 different tissue cores per TMA, were prepared using standard techniques described previously [46]. Experienced pathologists evaluated hematoxylin- and eosin-stained sections of HCC tissues and selected representative areas. One core from each tumor was included in the final TMA. The TNM classification system was used to define pathological primary tumor extent (pT stage) and disease stage according to the 8th edition of the Union for International Cancer Control (UICC) staging system [47].

Non-tumor liver tissues of 10 additional HBV (8 men and 2 women) and 9 additional HCV (7 men and 2 women)-infected patients were used for immunoblot analysis (Figure 1). These patients were also from the University Hospital Regensburg. The mean age of the HBV patients was 56.0 ± 16.3 years, fibrosis score was 2.3 ± 1.6 and inflammation score 2.8 ± 1.6. The age of the HCV patients was 56.4 ± 7.7 years, the fibrosis score was 3.8 ± 0.4 and the inflammation score was 2.2 ± 1.9. HCV patients had a higher fibrosis score than HBV patients (*p* = 0.023). Liver tissue of 6 patients (3 females and 3 males) of whom 3 had colon carcinoma, 1 hemangioma and 2 rectum carcinoma was also analyzed. The median age was 55.3 ± 9.7 years. These 6 liver-healthy patients had similar age and sex distribution in comparison to HBV and HCV patients. Moreover, non-tumor tissues of 6 patients with HBV (1 female, age: 62.2 ± 9.5 years, fibrosis score: 2.7 ± 1.4, inflammation score: 2.7 ± 1.5) and 6 patients with HCV (2 females, age: 55.3 ± 6.2 years, fibrosis score: 3.8 ± 0.4, inflammation score: 2.2 ± 2.0) were used for immunoblot analysis (Figure 1). The sex distribution, age, fibrosis and inflammation scores of these cohorts were similar.

Moreover, tumor and non-tumor tissues of 14 male patients with non-alcoholic fatty liver disease-caused HCC (age: 63.2 ± 9.6 years, fibrosis score: 2.7 ± 1.4, inflammation score: 2.7 ± 1.5) were used for immunoblot analysis (Figure 1).

### 2.2. Immunohistochemistry

The BAMBI antibody (order number: HPA010866) was obtained from Merck (Darmstadt, Germany). For immunohistochemistry, 4 μm thick sections of the TMA blocks were deparaffinized and treated at 120 °C for 5 min with Tris-EDTA buffer (pH 9). Peroxidase blocking solution (Dako, Glostrup, Denmark) was used for blocking endogenous peroxidase and incubation with the antibody was performed for 30 min at room temperature. Staining was performed by the Dako EnVision™^+^ Detection System, Peroxidase/DAB+, anti-mouse/anti-rabbit (Dako, Glostrup, Denmark). Finally, the slides were counterstained with hematoxylin.

A three-stage scoring system was used for BAMBI, with a score of 1 indicating low cytoplasmic and/or membranous staining intensity, 2 indicating moderate staining intensity, and 3 indicating high staining intensity in >80% of hepatocytes.

### 2.3. Histological Scores

Histological assessment of the grade of inflammation and liver fibrosis was performed by the experienced liver pathologists K.U. and K.E. Intratumoral inflammation was scored on a 4-point scale from 0 to 3, with 0 indicating no inflammation and 3 indicating severe inflammation. Fibrosis of liver parenchyma in the surgical specimen was graded according to the Ishak fibrosis score on a 7-point scale from 0 to 6, where 0 represents no fibrosis and 6 represents cirrhosis [48].

### 2.4. Immunoblot

Immunoblot was performed as described [49]. Liver tissues were solubilized in radioimmunoprecipitation assay lysis buffer (50 mM Tris-HCl, pH 7.4, 150 mM NaCl, 5 mM EDTA, 0.05% *v*/*v* Nonidet P-40, 1% *v*/*v* sodium deoxycholate, 1% *v*/*v* Triton X-100 and 0.1% *v*/*v* SDS). A total of 20 µg of protein was separated using sodium dodecyl-sulfate polyacrylamide gel electrophoresis (nUView Tris-Glycine SDS-PAGE Precast Gel 4–20%; Biotrend Chemikalien GmbH, Cologne, Germany) and the proteins were transferred to polyvinylidene difluoride membranes (Bio-Rad, Feldkirchen, Germany). Antibody incubations were carried out overnight in phosphate-buffered saline containing 0.1% Tween and 1% BSA. The enhanced chemiluminescence Western blot detection system (Amersham Pharmacia, Deisenhofen, Germany) was used for immunodetection. The BAMBI antibody was from ThermoFisher Scientific (Waltham, MA, USA; order number: BS-12418R) and GAPDH antibody was from New England Biolabs GmbH (Frankfurt, Germany). The dilution of the antibodies was 1:1000.

### 2.5. Statistics

Data are given as mean value ± standard deviation (SD). Statistical tests used were the Wilcoxon Test, Mann–Whitney U Test and Kruskal–Wallis Test (SPSS Statistics 26.0 program). For calculations of the pT categories, these were scored as follows: pT1a as 1, pT1b as 2, pT2 as 3, pT3 as 4 and pT4 as 5. A *p*-value < 0.05 was considered significant.

## 3. Results

### 3.1. BAMBI Expression in HCC Tissues

BAMBI protein levels were analyzed by immunohistochemistry and the expression in hepatocytes was scored using a three-stage scoring system, with a score of 1 indicating low cytoplasmic and/or membranous staining intensity, 2 indicating moderate staining intensity and 3 indicating high staining intensity in >80% of hepatocytes.

**Figure 2:**
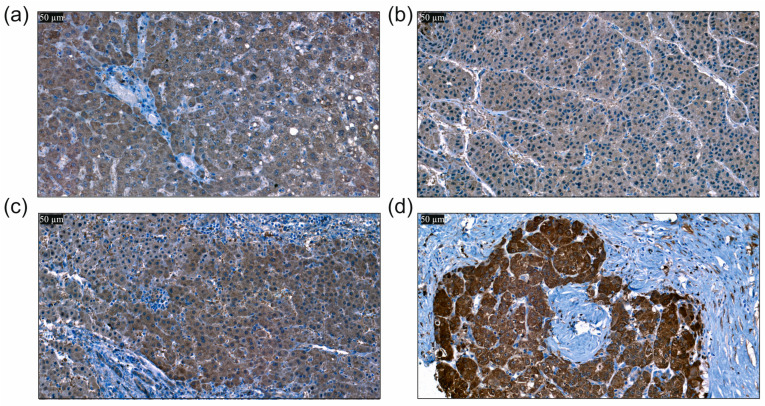
Immunohistochemical expression of BAMBI in hepatocellular carcinoma (HCC). The slides were counterstained with hematoxylin after staining with the antibody. The brown staining shows the expression of BAMBI protein in hepatocytes, which was scored. (**a**) Immunohistochemical expression of BAMBI in non-neoplastic liver tissue of a patient with HCC. (**b**) Immunohistochemical expression of BAMBI in HCC tissue with staining of hepatocytes scored as 1 for weak staining. (**c**) Immunohistochemical expression of BAMBI in HCC tissue with staining of hepatocytes scored as 2 for moderate staining. (**d**) Immunohistochemical expression of BAMBI in HCC tissue with staining of hepatocytes scored as 3 for strong staining (400-fold magnification in all images; scale bar: 50 µm).

BAMBI protein was low expressed in 48%, moderately expressed in 42% and high expressed in 10% of the 320 HCC tissues analyzed (Figure 2).

Stratification into low, moderate and high BAMBI-expressing tumors showed that the T stage, lymph node invasion, blood vessel invasion, histologic grading and tumor size did not differ between patients with low, moderate or high BAMBI levels. Accordingly, the Union for International Cancer Control (UICC) stage of the patients stratified for BAMBI protein was comparable. Age did not differ between the three groups (Table 1). BAMBI protein was not associated with inflammation or fibrosis grade (Table 1).

### 3.2. BAMBI Expression in Non-Tumor and Tumor Tissues of Patients with HCC

BAMBI protein analyzed by immunohistochemistry of HCC tissues was 1.6 ± 0.7 and of non-tumor tissues was 1.5 ± 0.6, and was similar (*p* = 0.164) between these tissues.

BAMBI protein in normal tissues of females and males was similar (*p* = 0.378). BAMBI protein in the non-tumor tissues was not correlated with fibrosis and inflammation scores (*p* > 0.05).

**Figure 3:**
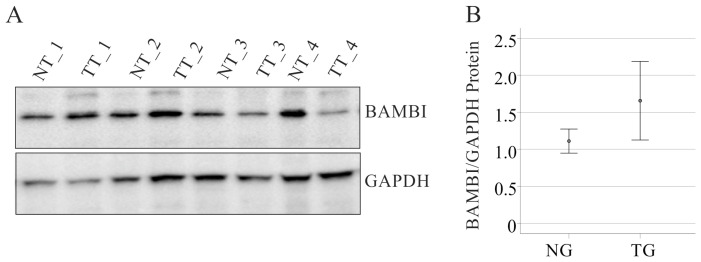
Immunoblot of BAMBI protein in non-tumor and tumor tissues of patients with HCC. (**A**) Immunoblot of BAMBI protein in paired non-tumor (NT) and tumor tissues (TT) of four patients with HCC. GAPDH was used as loading control; (**B**) BAMBI protein expression normalized to GAPDH protein, which were both quantified by ImageJ [50] (https://imagej.net/ij/) of 14 patients.

To further evaluate the normal expression of BAMBI protein in HCC, this was also analyzed by immunoblot in paired non-tumor and tumor tissues of 14 patients with non-viral HCC. This showed that BAMBI protein was similar in non-tumor and tumor tissues (Figure 3A). BAMBI levels normalized to GAPDH were 1.1 ± 0.6 in non-tumor tissue and were 1.7 ± 2.0 (*p* = 0.198) in tumors (Figure 3B).

### 3.3. BAMBI Expression in HCC Tissues of Women and Men

Women and men had similar age (*p* = 0.331). BAMBI staining scores of the HCC tissues of women and men did not differ (*p* = 0.171). T stage (*p* = 0.620), lymph node invasion (*p* = 0.454), vessel invasion (*p* = 0.965), and UICC score (*p* = 0.459) were similar between sexes. Tumors of females were 8.28 ± 5.98 cm and of males 6.19 ± 4.53 cm; so, they were larger in females (*p* = 0.022).

A sex-specific analysis showed that the BAMBI protein levels of female HCC tissues were not related to T stage, lymph node invasion, vessel invasion, grading, tumor size, or UICC score (Table 2). BAMBI protein in the HCC tissues of women was not related to age, inflammation, or fibrosis grade (Table 2).

BAMBI protein levels of male HCC tissues were not related to T stage, lymph node invasion, vessel invasion, grading, tumor size, or UICC score (Table 2). BAMBI protein in men’s HCC tissue had no association with age, inflammation or grade of fibrosis (Table 2).

### 3.4. BAMBI Expression in HCC Tissues of HBV- and HCV-Infected Patients

Disease etiology was documented for 47 patients; 15 patients had HBV (2 females) and 32 patients (8 females) had HCV. HBV and HCV patients had similar sex distribution, age, T stage, lymph node invasion, grading, tumor size and UICC score (*p* > 0.05 for all). Vessel invasion (*p* = 0.012) of HCV HCC tissues was less and inflammation (*p* = 0.023) was increased in comparison to HCC tissues of HBV patients. Fibrosis grade did not differ between HBV and HCV infected patients (*p* = 0.684).

**Figure 4:**
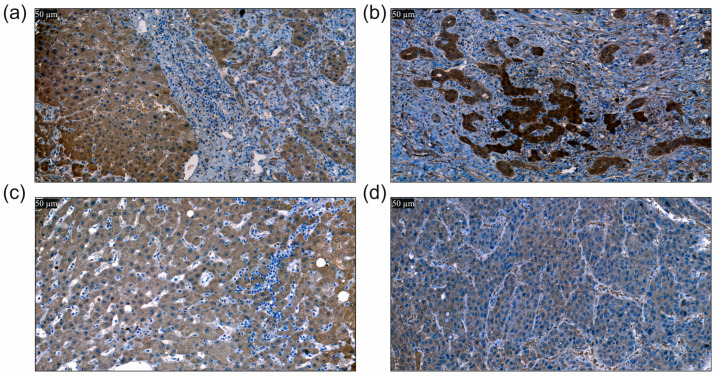
Immunohistochemical expression of BAMBI in non-tumor and tumor tissues of HBV and HCV-infected patients. The slides were counterstained with hematoxylin after staining with the antibody. The brown staining shows the expression of BAMBI protein in hepatocytes, which was scored. (**a**) Immunohistochemical expression of BAMBI in non-neoplastic liver tissue of a patient with HBV and HCC. (**b**) Immunohistochemical expression of BAMBI in HCC tissue of a patient with HBV. (**c**) Immunohistochemical expression of BAMBI in non-neoplastic liver tissue of a patient with HCV and HCC. (**d**) Immunohistochemical expression of BAMBI in HCC tissue of a patient with HCV (400-fold magnification in all images; scale bar: 50 µm).

BAMBI protein expression of HBV tumors was significantly higher (*p* = 0.006) with BAMBI protein levels of 1.9 ± 0.6 in HBV compared to 1.3 ± 0.5 in HCV (*p* = 0.006). Representative pictures of the tumor tissues of HBV- and HCV-infected patients are shown in Figure 4.

In the HCV cohort, the 22 patients with low BAMBI expression had a hepatic inflammation score of 0.74 ± 0.56 and those 10 patients with moderate BAMBI levels had a hepatic inflammation score of 1.55 ± 0.73 (*p* = 0.006). All other parameters were similar between patients with low and moderate BAMBI levels. The HBV cohort was too small for such a calculation.

### 3.5. BAMBI Expression in Non-Tumorous Tissues of HBV- and HCV-Infected Patients

**Figure 5:**
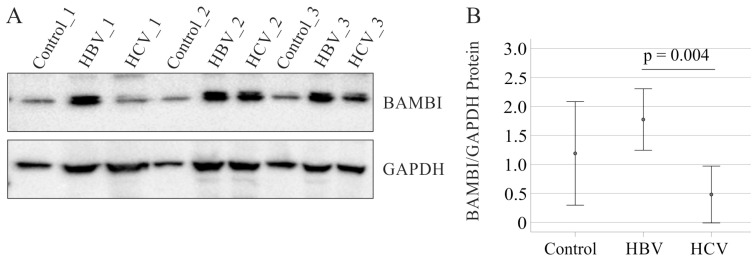
Immunoblot of BAMBI protein in liver tissues of patients without HCC (controls), and in non-tumor tissues of HBV and HCV infected patients. (**A**) BAMBI protein in the liver of three controls (Control_1, Control_2, Control_3), non-tumor tissues of three HBV (HBV_1, HBV_2, HBV_3)- and non-tumor tissues of three HCV (HCV_1, HCV_2, HCV_3)-infected patients. GAPDH was used as loading control. (**B**) BAMBI protein expression normalized to GAPDH protein, which were both quantified by ImageJ [50] (https://imagej.net/ij/) of 10 HBV, 9 HCV and 6 normal liver tissues.

To evaluate whether BAMBI is also low expressed in non-tumorous liver tissues of HCV- compared to HBV-infected patients, immunoblot analysis was performed. The immunoblot method has the advantage that the protein levels can be quantified whereas immunohistochemistry is a semiquantitative approach. BAMBI protein was significantly lower in the non-HCC tissues of HCV compared to HBV-infected patients. The differences between liver-healthy controls and HBV and between liver-healthy controls and HCV were not significant (Figure 5).

In addition, non-tumor tissues of additional patients with HBV and HCV (six patients per group) were compared (Figure S1A,B). This experiment showed lower BAMBI protein in HCV. Analysis of microarray expression data (GEO accession number for the data GSE20948) from JFH-1 (a genotype 2a HCV clone)-infected Huh7 cells [51] showed downregulation of *BAMBI* mRNA levels in the infected cells at 12, 18, 24 and 48 h compared to mock infected cells (Figure S1C).

### 3.6. BAMBI Expression and Survival

The mean overall survival of patients with low tumor levels of BAMBI was 4.9 ± 0.4 years, with moderate BAMBI levels was 5.0 ± 0.4 years and with strong BAMBI levels was 5.6 ± 1.1 years and did not differ between these groups (*p* = 0.806). Metastasis-free survival (*p* = 0.787) and recurrence-free survival (*p* = 0.821) were not associated with hepatocyte BAMBI levels.

## 4. Discussion

The current study showed normal BAMBI protein expression in HCC tissues, which was not associated with disease progression and survival. BAMBI protein was expressed at low levels in normal and HCC tissues from patients with chronic HCV compared to patients with HBV, who had normal BAMBI protein levels.

Similar levels of BAMBI protein in HCC and non-tumor liver tissues are in contrast to a study from China, where tumor BAMBI protein levels were found to be elevated [21]. A previous study from our group analyzed tumor-adjacent and HCC expression of BAMBI by immunoblot and observed low BAMBI protein in the tumors. This analysis included very few patients, which is a limitation of this study [10].

Ethnic differences in HCC have been described between Asians and Europeans [44,45], but whether this also applies to BAMBI requires further analysis. Chronic HBV is the cause of almost 80% of HCC in Asia, but is less common in Europe [42,43]. Paired tumor and non-tumor tissues from patients with HBV, which were not available for our study, are needed to clarify whether upregulation of the BAMBI protein in HCC is more common in these patients.

In the current work, we also performed immunoblotting of 14 paired tumor and non-tumor tissues from patients with HCC. Again, the BAMBI protein of these tissues was similar. While immunoblot analysis detects total BAMBI protein levels in the tissues, in immunohistochemistry we only assessed BAMBI protein expression in hepatocytes. However, hepatocytes are the major cell population in the liver [52] and account for most of the BAMBI protein in cell lysates. BAMBI is not expressed in Kupffer cells and BAMBI protein levels in primary human hepatocytes are much higher than in hepatic stellate cells [10]. This indicates that the BAMBI signal from the immunoblot is very similar to the expression in hepatocytes.

BMPs promote malignant phenotypes in several tumor types, and the expression of BMP4 and BMP9 is induced in HCC [36,40,53]. Strong upregulation of BAMBI in HCC, as suggested by previous analyses [21] is thought to block BMP signaling [2]. The normal expression of BAMBI in HCC tissues according to our data is consistent with higher BMP activities in HCC tissues [36,37] because the relative levels of BMPs to BAMBI are increased. However, this is a very simplified view of the complex pathways that regulate BMP signaling [3,40,53].

Consistent with the normal expression of BAMBI in HCC tissues, we did not find an association of BAMBI protein in HCC tissues with tumor size, tumor stage, lymph node and vascular invasion in our comparatively large cohort. Sex-specific analysis showed similar results, also excluding any correlation of BAMBI protein in HCC tissue with HCC progression.

Interestingly, immunohistochemistry showed that HCV infection was associated with lower BAMBI protein levels in HCC tissues in comparison to HCC tissues of patients with HBV infection. Immunoblot experiments using non-tumorous liver tissues of liver-healthy controls, HBV and HCV patients detected lower BAMBI protein in HCV in comparison to HBV infection.

Analysis of microarray expression data from HCV-infected Huh7 cells [51] showed a downregulation of *BAMBI* mRNA levels in the infected cells. This experiment further supports a direct effect of HCV infection on hepatocyte *BAMBI* expression, although the underlying mechanisms and the pathophysiological relevance are unclear. Moreover, analysis of BAMBI protein was not performed in these cells [51].

HBV and HCV patients, of whom tissues were used for immunohistochemistry, had similar T stage, lymph node invasion, grading, tumor size and UICC score. Vessel invasion of HCV HCC tissues was less and inflammation was increased in comparison to HCC tissues of HBV patients. Fibrosis grade did not differ between HBV- and HCV-infected patients. BAMBI protein was not correlated with vessel invasion and was positively associated with inflammation in HCV showing that low BAMBI protein levels in HCV are not associated with these features.

Variable protein expression in HCC tissues of HBV and HCV patients has been shown before. The expression of superoxide dismutase (Cu/Zn) showed a decrease in HBV- but not HCV-related HCC. Heat shock protein was decreased in HBV and increased in HCV HCC tissue in comparison to non-tumor tissues. However, this study did not compare non-tumor tissues of HBV- and HCV-infected livers [54] and whether these proteins also differ in the non-tumor tissues of these patients has still to be clarified. A comparison of BMP4 activities in HCC tissues from HBV- and HCV-infected tissues has not been carried out to our knowledge.

The Wnt/beta-catenin signaling pathway is a tumor-promoting factor in HCC [2] and overexpression occurs more frequently in HCV- than HBV-related HCCs [55]. However, whether BAMBI is regulated by this pathway has not been resolved [2,19]. Moreover, studies suggest that BAMBI induces the activation of the Wnt/beta catenin pathway [56,57]. To further evaluate an association of BAMBI protein levels with the Wnt/beta catenin pathway in HCV, parallel analysis of these proteins in HCC tissues is required.

Notably, BAMBI protein in HCV was positively associated with liver inflammation whereas no associations with HCC status or fibrosis scores could be identified. Lipopolysaccharide was shown to downregulate BAMBI in hepatic stellate cells [9] but the effect of inflammatory mediators on hepatocyte BAMBI expression, which has been scored in our tissues, is less clear. Currently, we cannot explain the positive association of BAMBI protein with inflammation in HCC tissues, which was only observed in HCV.

A recent study has demonstrated higher expression of the angiotensin-converting enzyme 2 (ACE2) and increased infection with SARS-CoV-2 upon BAMBI knock-down in Huh7 cells [58]. HCV infection was shown to increase the expression of ACE2 in Huh7.5 cells [59]. Thus, it is reasonable to suggest that downregulation of BAMBI in HCV infection contributes to higher ACE2 levels of these patients.

Confounding factors in observational analysis are sex and age. It was observed that there were notable differences in the incidence of HCC between men and women. This disparity may be attributed to a combination of environmental and biological factors [13] and variability in the HCC proteome [60]. However, BAMBI protein expression in males and females was similar in HCC and non-HCC tissues and did not correlate with the age of the patients.

This analysis has limitations. The disease etiology of most patients was unknown. Moreover, the descriptive nature of this retrospective study, where BAMBI protein was semi-quantified by a pathologist’s visual scoring of the staining intensity is a further limitation.

## 5. Conclusions

This study shows that BAMBI protein was equally expressed in non-tumor and HCC tissues and was not associated with liver disease and HCC severity. Accordingly, BAMBI levels were not related to survival. BAMBI protein levels in non-tumor and HCC tissue of patients with HCV were low compared to HBV infection, showing that disease etiology has to be considered when studying the role of BAMBI in fibrogenesis and carcinogenesis.

## Figures and Tables

**Table 1:** T stage, lymph node invasion, vessel invasion, grading, UICC stage, inflammation, fibrosis stages and age of patients stratified for low, moderate and high BAMBI protein levels in the tumors. There were no significant differences between these groups.

	Low BAMBI	Moderate BAMBI	High BAMBI
Patients	154	135	31
T stage	2.88 ± 0.99	2.86 ± 1.02	2.77 ± 1.23
Lymph node invasion	0.09 ± 0.29	0.07 ± 0.25	0.03 ± 0.18
Vessel invasion	0.61 ± 0.64	0.48 ± 0.62	0.48 ± 0.68
Grading	1.94 ± 0.69	1.95 ± 0.71	2.00 ± 0.68
Tumor size (cm)	6.97 ± 5.11	6.29 ± 4.71	5.84 ± 4.38
UICC score	1.97 ± 0.85	1.97 ± 0.92	1.93 ± 0.93
Inflammation grade	0.72 ± 0.64	0.78 ± 0.67	0.77 ± 0.82
Fibrosis grade	3.76 ± 2.49	4.08 ± 2.50	4.04 ± 2.50
Age (years)	64.8 ± 10.3	64.0 ± 13.0	63.90 ± 8.7

**Table 2:** T stage, lymph node invasion, vascular invasion, grading, UICC stage, inflammation, fibrosis stages and age of female and male patients stratified for low, moderate and high BAMBI protein levels in the tumors (inv, invasion).

	Low BAMBI	Moderate BAMBI	High BAMBI	Low BAMBI	Moderate BAMBI	High BAMBI
	Female Patients			Male Patients		
Patients	33	20	5	121	115	26
T stage	2.75 ± 0.92	3.00 ± 1.17	2.60 ± 1.34	2.92 ± 1.02	2.83 ± 1.00	2.81 ± 1.23
Lymph node inv	0.03 ± 0.17	0.05 ± 0.22	0.20 ± 0.45	0.11 ± 0.31	0.07 ± 0.26	0.0 ± 0.0
Vessel inv	0.58 ± 0.71	0.60 ± 0.82	0.40 ± 0.55	0.62 ± 0.62	0.46 ± 0.58	0.50 ± 0.71
Grading	1.94 ± 0.66	2.20 ± 0.77	2.20 ± 0.45	1.94 ± 0.69	1.89 ± 0.69	2.00 ± 0.69
Tumor size (cm)	7.92 ± 6.33	8.66 ± 5.69	9.20 ± 5.70	6.71 ± 4.72	5.89 ± 4.41	5.17 ± 3.87
UICC score	1.84 ± 0.85	2.00 ± 0.97	1.80 ± 1.10	2.00 ± 0.85	1.97 ± 0.92	1.96 ± 0.92
Inflammation grade	0.84 ± 0.69	0.79 ± 0.92	0.60 ± 0.55	0.69 ± 0.62	0.77 ± 0.63	0.80 ± 0.87
Fibrosis grade	3.08 ± 2.48	4.00 ± 2.54	3.60 ± 3.29	3.92 ± 2.47	4.09 ± 2.25	4.13 ± 2.38
Age (years)	64.2 ± 11.2	63.2 ± 12.4	57.6 ± 5.8	65.0 ± 10.1	64.2 ± 13.2	65.2 ± 8.7

## Data Availability

The data presented in this study are available on request from the corresponding author(s).

## Supplementary Materials

The following supporting information can be downloaded at: https://www.mdpi.com/article/10.3390/biomedicines12102397/s1, Figure S1: Immunoblot of BAMBI protein in non-tumor tissues of HBV and HCV infected patients and BAMBI mRNA in HCV-infected Huh7 cells.
