# Supplementary material for: Hepatic Bone Morphogenetic Protein and Activin Membrane-Bound Inhibitor Levels Decline in Hepatitis C but Are Not Associated with Progression of Hepatocellular Carcinoma
Source: Biomedicines. 2024 Oct 19;12(10):2397. doi: 10.3390/biomedicines12102397 (PMC11504530; doi:10.3390/biomedicines12102397)
Supplement: Supplementary file 1 [file biomedicines-12-02397-s001.zip › biomedicines-3250610-supplementary.pdf]

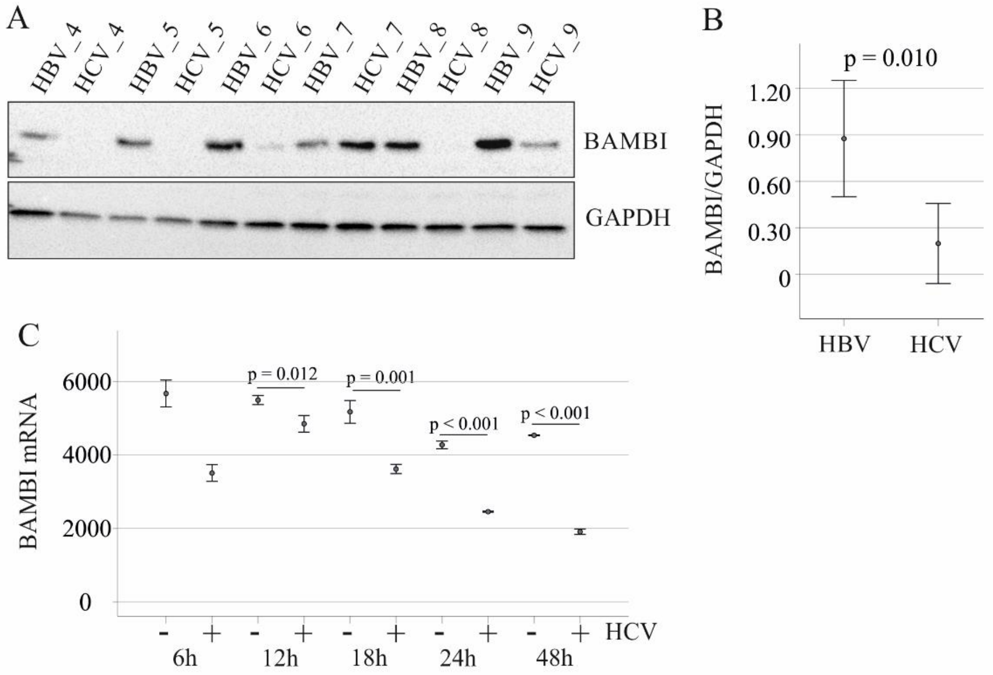

Figure S1. Immunoblot of BAMBI protein in non-tumor tissues of HBV and HCV infected patients and BAMBI mRNA in HCV-infected Huh7 cells. (**a**) BAMBI protein in the non-tumor tissues of 6 HBV and non-tumor tissues of 6 HCV infected patients. GAPDH was used as loading control; (**b**) BAMBI protein expression normalized to GAPDH protein levels, which were quantified by ImageJ, of the immunoblot shown in (a); (**c**) BAMBI mRNA levels of HCV infected Huh7 cells (+ HCV) at 6, 12, 18, 24 and 48 h after infection in comparison to mock infected cells (- HVC) (Microarray expression data GEO accession number GSE20948).
